# Phylogenetic Classification of Seed Plants of Taiwan

**DOI:** 10.1186/s40529-017-0206-6

**Published:** 2017-11-21

**Authors:** Cheng-Tao Lin, Kuo-Fang Chung

**Affiliations:** 1 Department of Biological Resources, National Chiayi University, Chiayi, 60004 Taiwan; 2 Research Museum and Herbarium (HAST), Biodiversity Research Center, Academia Sinica, Taipei, 11529 Taiwan

**Keywords:** Angiosperm Phylogeny Group classification, APG IV, Big new biology, Data cleaning, Flowering plants, Gymnosperms, Spermatophytina, TaiBIF, TaiCOL

## Abstract

**Background:**

Biological classification, the hierarchical arrangement of scientific names of organisms, constitutes the core infrastructure of biological databases. For an efficient management of biological databases, adopting a stable and universal biological classification system is crucial. Currently in Taiwan Biodiversity Information Facility (TaiBIF; http://taibif.tw/), the national portal website that integrates Taiwan’s biodiversity information databases, angiosperms are arranged according to Cronquist’s System of Classification, which is not compatible with current trend of the Angiosperm Phylogeny Group (APG) classification. To consolidate the function and management of the database, TaiBIF is moving to adopt the APG IV classification and Christenhusz et al. (Phytotaxa 19:55–70, 2011)’s classification of gymnosperms, which we summarize as the Phylogenetic Classification of Seed Plants of Taiwan.

**Results:**

The Phylogenetic Classification of Seed Plants of Taiwan places gymnosperms in five families [vs. eight families in the Flora of Taiwan (FOT)] and angiosperms in 210 families (vs. 193 families in FOT). Three FOT gymnosperm families are synonymized in current treatment. Of the 210 APG IV families, familial circumscriptions of 114 families are identical with FOT and 50 families are recircumscription of FOT, with 46 families newly added. Of the 29 FOT families not included in current classification, two families are excluded and 27 families are synonymized.

**Conclusions:**

The adoption of the Phylogenetic Classification of Seed Plants of Taiwan in TaiBIF will provide better service and efficient management of the nation’s biodiversity information databases.

**Electronic supplementary material:**

The online version of this article (10.1186/s40529-017-0206-6) contains supplementary material, which is available to authorized users.

## Background

Biological classification, the hierarchical arrangement of scientific names of organisms, provides keywords and links to catalogue and organize biological information (Patterson et al. 2014). Biological classification constitutes the core infrastructure of biological databases (Patterson et al. 2010, 2014). Adopting a stable and universal biological classification system not only is crucial for the users but also fundamental for the efficient management of the databases.

TaiBIF (Taiwan Biodiversity Information Facility; http://taibif.tw/) is the national portal website that integrates Taiwan’s biodiversity information (Shao et al. 2013) through TaiCOL (Catalogue of Life in Taiwan; http://col.taibif.tw/), TaiEOL (Taiwan Encyclopedia of Life; http://eol.taibif.tw/), TaiBOL (Cryobanking Program for Wildlife Genetic Material in Taiwan; http://cryobank.museum.biodiv.tw/), and TELDAP (Taiwan e-Learning and Digital Archives Programs; http://core.teldap.tw/). As an associate participant of GBIF (Global Biodiversity Information Facility; http://www.gbif.org/), TaiBIF also functions as a national node of GBIF (Shao et al. 2013). The initiation of TaiBIF started in 2003 with the establishment of TaiBNET (Taiwan Biodiversity National Information Network; http://taibnet.sinica.edu.tw), providing “Taiwan species checklist” and the list of local taxonomic experts (Shao et al. 2013). Currently in TaiCOL, the successor of TaiBNET, the flowering plants are arranged according to Cronquist (1968)’s System of Classification (Shao et al. 2008), replacing A. Engler’s *Syllabus der Pflanzenfamilien* that was adopted in the Flora of Taiwan (FOT), 2nd edition (Huang 1994). Although Cronquist’s System was highly influential and had been followed by several major floras such as Flora of North America (Reveal 1993) and Flora of Australia (Kanis et al. 1999), much of the content of Cronquist System is not compatible to the current trend of the APG classification.

The Angiosperm Phylogeny Group (APG) classification of the orders and families of flowering plants, now in its fourth edition (APG IV), is a collaborative effort of plant molecular systematic community worldwide (The Angiosperm Phylogeny Group 1998, 2003, 2009, 2016), providing the greatest stability and predictability regarding biodiversity information of flowering plants (Mayr 1981; Wearn et al. 2013). Although APG classification has not been adopted officially in Taiwan, families circumscribed by molecular phylogenetic studies and summarized by APG have been increasingly accepted by both academic (Hsu et al. 2011, 2016a, b; Wu et al. 2015) and citizen scientists (e.g., Nature Campus http://nc.biodiv.tw/bbs/index.php).

As an official provider of biodiversity information of the country, the classification systems followed by TaiCOL has deep and profound influences. In an effort to consolidate the function and management of TaiBIF that shall result in stable and better services of the websites, it is inevitable for TaiCOL to adopt classification systems that are constructed based on results of robust molecular phylogenetic analyses. This article outlines phylogenetic classification of families of the seed plants of Taiwan summarized based on Christenhusz et al. (2011)’s classification of gymnosperms, APG IV, and subsequent studies. To facilitate the transition toward APG IV, we also provide the spreadsheet of the classification schema for all seed plant genera that will be adopted by TaiCOL (Additional file 1: Appendix S1). This spreadsheet will be updated constantly and can be downloaded through TaiCOL. A brief note is provided for families of which circumscription has been changed between the treatment of FOT and APG IV classification.

## Methods

The database of seed plants of Taiwan was compiled from “a checklist of the vascular plants of Taiwan” of the Flora of Taiwan (Boufford et al. 2003), “Illustrated Guide to Aquatic Plants of Taiwan” (Yang et al. 2001), Wu et al. (2010) that summarized naturalized and invasive flora, subsequently published native (e.g., Hsu et al. 2011; Wu et al. 2015) and naturalized (e.g., Liang et al. 2011; Wang et al. 2016) species, and the flora of Tongsha (Pratas) Island (Huang et al. 1994; Lin et al. 2005). The checklist was then imported into relational PostgreSQL database as a basis for migrating process. The migration process applied a ‘data cleaning framework’ to improve our data set quality through diagnosing, detecting, and correcting procedures. The data cleaning procedure included three major stages: (1) error type defining, (2) error instance identifying, and (3) error correcting (Maletic and Marcus 2000). Furthermore, we followed the data cleaning principles and methods suggested by Chapman (2005) when processing nomenclature data. In the initial stage of migration, instead of constructing a name-based database, a taxon-based database, which includes a unique taxonomy identifier (taxon ID) and several attributes such as family, genus, scientific names and vernacular names, etc., was constructed. In order to reduce the redundancy of the database and improve the data quality and integrity, we adopted relational database normalization to parse the raw data table into a second normal form schema. Through the normalization process, potential errors such as duplicate entries, misplaced taxa, etc., could be eliminated efficiently. In the second stage, we automated a python script to cross-validate our data base with Missouri Botanical Garden’s Tropicos (http://www.tropicos.org/) and International Plant Names Index (IPNI, http://ipni.org), identifying unmatched or unfound names for manual checking. In the third stage, three major possible errors or problems: (1) illegitimate or invalid names, (2) misspelled names, (3) different taxonomic treatment, were corrected after cross-validation.

We adopted Ruggiero et al. (2015) for the higher level classification of seed plants (Subphylum Spermatophytina and above). For gymnosperms (Superclass Gymnospermae), Christenhusz et al. (2011)’s classification was followed, though caution was taken for the uncertainty of the phylogenetic position of gnetophytes (Lu et al. 2014; Wang and Ran 2014). For angiosperms (Superclass Angiospermae), major clades recognized as superorders in Chase and Reveal (2009) and the classification of The Angiosperm Phylogeny Group (2016) was adopted, with the exception of Boraginales in which Luebert et al. (2016)’s new familial classification was followed. For orders and families of which vernacular names are lacking in the current literature of the flora of Taiwan, the names proposed by Liu et al. (2015) were followed.

## Results and discussion

**Fig. 1:**
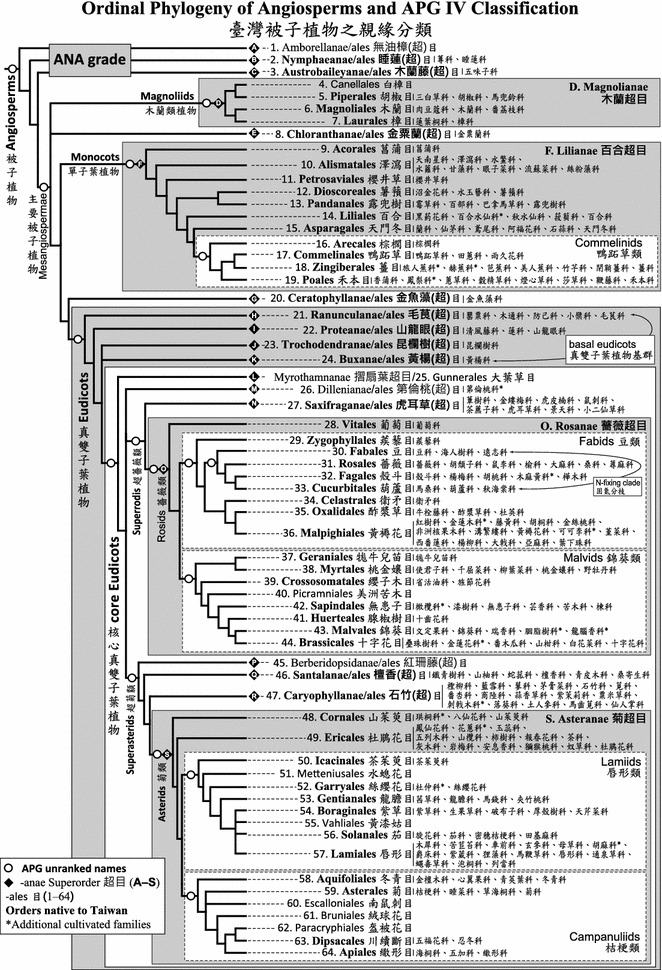
Ordinal phylogeny of angiosperms and APG IV classification, with notes on familial classification of angiosperm families of Taiwan

Based on Christenhusz et al. (2011), APG IV (2016), and Luebert et al. (2016)’s familial classification of Boraginales, the “Phylogenetic Classification of Seed Plants of Taiwan” is presented below. Of the four classes (I–IV), eight orders (A–H), and 12 families of gymnosperms in Christenhusz et al. (2011)’s classification, five families in four orders of two classes are naturally distributed in Taiwan. Compared to the treatment in FOT, current classification includes Taxodiaceae in Cupressaceae and expands Taxaceae to include Amentotaxaceae and Cephalotaxaceae. Of the 16 superorders (A–S), 64 orders, and 427 families currently circumscribed (Luebert et al. 2016; APG IV 2016; Thulin et al. 2016), 210 families in 53 orders of 15 superorders are recorded in the flora of Taiwan (Fig. 1; Table 1).

**Table 1:** Current taxonomic status of the families recorded in the Flora of Taiwan (FOT), 2nd ed. (Boufford et al. 2003) and newly added families to the flora of Taiwan based on APG IV

FOT families excluded (2)	IIBa.43 Rafflesiaceae and IIBa.125 Hydrophyllaceae
FOT families synonymized (27)	IIBa.7A Cecropiaceae, IIBa.20 Chenopodiaceae, IIBa.26 Illiciaceae, IIBa.48A Fumariaceae, IIBa.70 Aceraceae, IIBa.71A Bretschneideraceae, IIBa.81 Leeaceae, IIBa.83 Tiliaceae, IIBa.85 Bombacaceae, IIBa.86 Sterculiaceae, IIBa.89 Flacourtiaceae, IIBa.101A Trapaceae, IIBa.103 Theligonaceae, IIBa.104 Alangiaceae, IIBa.109 Pyrolaceae, IIBa.111 Myrsinaceae, IIBa.122 Asclepiadaceae, IIBa.128 Callitrichaceae, IIBa.137 Myoporaceae, IIBa.140 Valerianaceae, IIBa.141 Dipsacaceae, IIBb.6 Zannichelliaceae, IIBb.8 Najadaceae, IIBb.11 Agavaceae, IIBb.28(A) Taccaceae, IIBb.30 Lemnaceae, and IIBb.33 Sparganiaceae
FOT families circumscription changed (50)	IIBa.3 Salicaceae, IIBa.6 Ulmaceae, IIBa.7 Moraceae, IIBa.8 Urticaceae, IIBa.9A Olacaceae, IIBa.10 Santalaceae, IIBa.11 Loranthaceae, IIBa.14 Phytolaccaceae, IIBa.18 Portulacaceae, IIBa.21 Amaranthaceae, IIBa.25 Schisandraceae, IIBa.45 Theaceae, IIBa.46 Clusiaceae (≡ Guttiferae), IIBa.48 Papaveraceae, IIBa.49 Capparaceae, IIBa.51 Hamamelidaceae, IIBa.53 Saxifragaceae, IIBa.61 Euphorbiaceae, IIBa.71 Sapindaceae, IIBa.75 Celastraceae, IIBa.78 Icacinaceae, IIBa.80 Vitaceae, IIBa.84 Malvaceae, IIBa.95 Lythraceae, IIBa.105 Cornaceae, IIBa.106 Araliaceae, IIBa.107 Apiaceae (≡ Umbelliferae), IIBa.110 Ericaceae, IIBa.112 Primulaceae, IIBa.119 Loganiaceae, IIBa.120 Gentianaceae, IIBa.121 Apocynaceae, IIBa.123 Rubiaceae, IIBa.126 Boraginaceae, IIBa.127 Verbenaceae, IIBa.129 Lamiaceae (≡ Labiatae), IIBa.131 Scrophulariaceae, IIBa.133 Acanthaceae, IIBa.135 Orobanchaceae, IIBa.138 Plantaginaceae, IIBa.139 Caprifoliaceae, IIBb.1 Alismataceae, IIBb.2 Hydrocharitaceae, IIBb.4 Potamogetonaceae, IIBb.9 Liliaceae, IIBb.12 Amaryllidaceae, IIBb.14 Dioscoreaceae, IIBb.29 Araceae, IIBb.33 Typhaceae, and IIBb.35 Zingiberaceae
FOT families circumscription unchanged (114)	IIBa.1 Myricaceae, IIBa.2 Juglandaceae, IIBa.4 Betulaceae, IIBa.5 Fagaceae, IIBa.9 Proteaceae, IIBa.10A, Opiliaceae, IIBa.12 Balanophoraceae, IIBa.13 Polygonaceae, IIBa.15 Nyctaginaceae, IIBa.16 Molluginaceae, IIBa.17 Aizoaceae, IIBa.18A Basellaceae, IIBa.19 Caryophyllaceae, IIBa.22 Magnoliaceae, IIBa.23 Annonaceae, IIBa.24 Myristicaceae, IIBa.27 Lauraceae, IIBa.28 Hernandiaceae, IIBa.29 Trochodendraceae, IIBa.30 Ranunculaceae, IIBa.31 Berberidaceae, IIBa.32 Lardizabalaceae, IIBa.33 Menispermaceae, IIBa.34 Nelumbonaceae, IIBa.35 Nymphaeaceae, IIBa.36 Cabombaceae, IIBa.38 Ceratophyllaceae, IIBa.39 Saururaceae, IIBa.40 Piperaceae, IIBa.41 Chloranthaceae, IIBa.42 Aristolochiaceae, IIBa.44 Actinidiaceae, IIBa.47 Droseraceae, IIBa.50 Brassicaceae (≡ Cruciferae), IIBa.52 Crassulaceae, IIBa.54 Pittosporaceae, IIBa.55 Rosaceae, IIBa.56 Connaraceae, IIBa.57 Fabaceae (≡ Leguminosae), IIBa.58 Oxalidaceae, IIBa.59 Geraniaceae, IIBa.60 Zygophyllaceae, IIBa.62 Daphniphyllaceae, IIBa.63 Rutaceae, IIBa.64 Simaroubaceae, IIBa.65 Meliaceae, IIBa.66 Malpighiaceae, IIBa.67 Polygalaceae, IIBa.68 Coriariaceae, IIBa.69 Anacardiaceae, IIBa.72 Sabiaceae, IIBa.73 Balsaminaceae, IIBa.74 Aquifoliaceae, IIBa.76 Staphyleaceae, IIBa.77 Buxaceae, IIBa.79 Rhamnaceae, IIBa.82 Elaeocarpaceae, IIBa.87 Thymelaeaceae, IIBa.88 Elaeagnaceae, IIBa.90 Violaceae, IIBa.91 Stachyuraceae, IIBa.91A Passifloraceae, IIBa.92 Elatinaceae, IIBa.93 Begoniaceae, IIBa.94 Cucurbitaceae, IIBa.96 Myrtaceae, IIBa.97 Lecythidaceae, IIBa.98 Melastomataceae, IIBa.99 Rhizophoraceae, IIBa.100 Combretaceae, IIBa.101 Onagraceae, IIBa.102 Haloragaceae, IIBa.108 Diapensiaceae, IIBa.113 Plumbaginaceae, IIBa.114 Sapotaceae, IIBa.115 Ebenaceae, IIBa.116 Styracaceae, IIBa.117 Symplocaceae, IIBa.118 Oleaceae, IIBa.124 Convolvulaceae, IIBa.130 Solanaceae, IIBa.132 Bignoniaceae, IIBa.134 Gesneriaceae, IIBa.136 Lentibulariaceae, IIBa.142 Campanulaceae, IIBa.142A Sphenocleaceae, IIBa.143 Goodeniaceae, IIBa.144 Asteraceae (≡ Compositae), IIBb.3 Aponogetonaceae, IIBb.5 Ruppiaceae, IIBb.7 Zosteraceae, IIBb.9A Petrosaviaceae, IIBb.10 Stemonaceae, IIBb.13 Hypoxidaceae, IIBb.15 Smilacaceae, IIBb.16 Pontederiaceae, IIBb.17 Iridaceae, IIBb.18 Burmanniaceae, IIBb.19 Philydraceae, IIBb.20 Juncaceae, IIBb.21 Commelinaceae, IIBb.22 Xyridaceae, IIBb.23 Eriocaulaceae, IIBb.24 Flagellariaceae, IIBb.25 Cyperaceae, IIBb.26 Poaceae (≡ Gramineae), IIBb.27 Arecaceae (≡ Palmae), IIBb.28 Cyclanthaceae, IIBb.31 Pandanaceae, IIBb.34 Musaceae, IIBb.36 Cannaceae, IIBb.37 Marantaceae, IIBb.38 Orchidaceae, and IIBb.39 Triuridaceae
Newly added families (46)	F9.27 Acoraceae, F10.41 Cymodoceaceae, F12.43 Nartheciaceae, F14.53 Melanthiaceae, F14.56 Colchicaceae, F15.72 Asphodelaceae, F15.74 Asparagaceae, F18.88 Costaceae, N27.123 Altingiaceae, N27.127 Iteaceae, N27.128 Grossulariaceae, O30.141 Surianaceae, O31.149 Cannabaceae, O36.184 Calophyllaceae, and O36.186 Hypericaceae, O36.189 Putranjivaceae, O36.208 Linaceae, O36.211 Phyllanthaceae, O41.234 Dipentodontaceae, O43.245 Muntingiaceae, O44.254 Akaniaceae, O44.257 Caricaceae, O44.269 Cleomaceae, Q46.278 Schoepfiaceae, R47.281 Tamaricaceae, R47.306 Petiveriaceae, R47.314 Talinaceae, R47.317 Cactaceae, S48.320 Hydrangeaceae, S49.332 Pentaphylacaceae, S49.345 Mitrastemonaceae, S52.351 Garryaceae, S54.357B Coldeniaceae, S54.357C Cordiaceae, S54.357D Ehretiaceae, S54.357E Heliotropiaceae, S56.363 Hydroleaceae, S57.373 Linderniaceae, S57.384 Mazaceae, S57.385 Phrymaceae, S57.386 Paulowniaceae, S58.388 Stemonuraceae, S58.389 Cardiopteridaceae, S58.391 Helwingiaceae, S59.400 Menyanthaceae, and S63.408 Adoxaceae
Additional cultivated families (20)	A.2 Zamiaceae, B.3 Ginkgoaceae, G.8 Araucariaceae, F14.55 Alstroemeriaceae, F18.82 Strelitziaceae, F18.84 Heliconiaceae, F19.91 Bromeliaceae, M26.120 Dilleniaceae, O32.156 Casuarinaceae, O36.181 Ochnaceae, O36.197 Chrysobalanaceae, O42.238 Burseraceae, O43.250 Bixaceae, O43.253 Dipterocarpacea, O44.255 Tropaeolaceae, R47.311 Didiereaceae, S48.318 Nyssaceae, S49.329 Polemoniaceae, S52.350 Eucommiaceae, and S57.376 Pedaliaceae

The adoption of APG IV affects 79 (64 in dicotyledons and 15 in monocotyledons) of the 193 families (152 in dicotyledons and 41 in monocotyledons) recorded in FOT (Boufford et al. 2003), including changes in familial recircumscriptions in 50 families (41 in dicotyledons and 9 in monocotyledons), synonymization of 27 families (21 in dicotyledons and 6 in monocotyledons), and two families not present in Taiwan (i.e., Rafflesiaceae and Hydrophyllaceae). Circumscriptions of 114 (59.1%) families [88 (57.9%) in dicotyledons and 26 (63.4%) in monocotyledons] of FOT remain unchanged. A total of 46 families are added under current classification. Table 1 summarizes current status of the families recorded in FOT (Boufford et al. 2003), newly added families (46), and frequently cultivated families not recorded in FOT (20).

The adoption of APG IV inevitably results in changes in statistics of the flora of Taiwan. For examples, Euphorbiaceae s.l. (89 species in 27 genera), Scrophulariaceae s.l. (73 species in 26 genera), and Liliaceae s.l. (48 species in 21 genera) were ranked as the 8th, 10th and 18th most species-rich families in FOT (Hsieh 2003); however, under APG IV classification, Euphorbiaceae reduces to ca. 60 species in 17 genera, Scrophulariaceae to only 4 species in 3 genera, and Liliaceae to ca. 8 species in 2 genera. Saxifragaceae is another case of drastic changes, reducing from 25 species in 13 genera to 7 species in 4 genera. On the other hand, families such as Malvaceae, Plantaginaceae, and Orobanchaceae expand greatly, increasing from eight, one, and four genera to 26, 16, and 14 genera, respectively.

In the Classification outlined below, codes composed of alphabet and number(s) are applied to each family to denote its ordinal (and superordinal) classification. For gymnosperms, Christenhusz et al. (2011)’s alphabetical (A–H) and numeric (1–12) codes for Orders and Families are adopted. For angiosperms, the numeric codes of APG IV families (1–416) are followed, with the addition of alphabetical (A–S) code for superorders and numerical (1–64) codes for orders modified from Chase and Reveal (2009). For example, “F14.60. Liliaceae 百合科” indicates Superorder Lilianae (F), Order Liliales (14), and Family Liliaceae (60). Code designations of superorders and orders are outlined in Fig. 1. The numeric family codes used in the Flora of Taiwan (Boufford et al. 2003) are also listed in parentheses after the Chinese vernacular family name to aid an easy comparison to families circumscribed in FOT. For examples, “(≡ IIA.1)” in Cycadaceae indicates Family 1 of Gymnosperma (IIA) in Boufford et al. (2003), “(≡ IIBa.35)” of Nymphaeaceae denotes Family 35 of Dicotylendons (IIBa), and “(≡ IIBb.7)” of Zosteraceae stands for Family 7 of Monocotyledons (IIBb), while the sign “≡” indicates unchanged familial circumscription between FOT and current treatment.

For those families of which circumscription has been changed, the number of genera in FOT and current treatment are also provided. For examples, “F14.60. Liliaceae 百合科 (IIBb.9; 21/2)” indicates 21 genera included in Family 9 of Monocotyledons (IIBb) in FOT, while only 2 genera are included in current classification. A statement is followed to denote newly added genera and/or genera excluded. For examples, current classification of “F18.89. Zingiberaceae 薑科 (IIBb.35; 5/5)” includes the genus *Curcuma* 薑黃屬 based on Wu et al. (2010), while the genus *Costus* of FOT is moved to F18.88 Costaceae, resulting in a total of five genera as recorded in FOT (5/5). For newly added families, genera included are listed with references to previous classification. For newly recorded families, the number in parentheses after the Chinese vernacular name indicate the number of genera included. For families whose circumscription remain unchanged (e.g., Lauraceae, Asteraceae, Fabaceae, Orchidaceae, etc.), the full list of genera included is summarized in Additional file 1: Appendix S1. The Chinese vernacular names for all scientific names of taxa are adopted from FOT and/or names proposed when published (e.g., Chung et al. 2010b; Hsu et al. 2011). For newly added taxa not published by Taiwanese authors, the Chinese names proposed by Liu et al. (2015) are adopted. Although not officially recorded as parts of the flora, current treatments of 21 frequently cultivated plant families in Taiwan not recorded in FOT (marked with *) are also included.

## Conclusions

### Phylogenetic Classification of Seed Plants of Taiwan

Kingdom Plantae 植物界, Subkingdom Viridiplantae 綠色植物亞界, Infrakingdom Streptophyta 鏈型植物次界, Superphylum Embryophyta 有胚植物超門, Phylum Tracheophyta 維管束植物門, Subphylum Spermatophytina 種子植物亞門 (Ruggiero et al. 2015)

- Superclass Gymnospermae 裸子植物超綱 (≡ gymnosperms in Chase and Reveal 2009 and Christenhusz et al. 2011)
  - Class I. Cycadopsida 蘇鐵綱, Subclass Cycadidae 蘇鐵亞綱
    - A. Cycadales 蘇鐵目
      - A.1. Cycadaceae 蘇鐵科 (≡ IIA.1)
      *A.2. Zamiaceae 藏米亞 (堅果鳳尾蕉) 科
  - Class II. Ginkgoopsida 銀杏綱, Subclass Ginkgooidae 銀杏亞綱
    - B. Ginkgoales 銀杏目
      *B.3. Ginkgoaceae 銀杏科
  - Class III. Gnetopsida 買麻藤綱, Subclass Gnetidae 買麻藤亞綱
    - C. Welwitschiales 二葉樹目
    - D. Gnetales 買麻藤目
    - E. Ephedrales 麻黃目
  - Class IV. Pinopsida 松綱, Subclass Pinidae 松亞綱
    - F. Pinales 松目
      - F.7. Pinaceae 松科 (≡ IIA.6)
    - G. Araucariales 南洋杉目
      *G.8. Araucariaceae 南洋杉科
      - G.9. Podocarpaceae 羅漢松科 (≡ IIA.5)
    - H. Cupressales 柏目
      - H.11. Cupressaceae 柏科 (IIA.8; 3/5)
        - Including IIA.7 Taxodiaceae (*Cryptomeria* 柳杉屬, *Cunninghamia* 杉木屬 and *Taiwania* 臺灣杉屬).
      - H.12. Taxaceae 紅豆杉科 (IIA.2; 1/3)
        - Including IIA.3 Amentotaxaceae (*Amentotaxus*穗花杉屬) and IIA.4 Cephalotaxaceae (*Cephalotaxus* 粗榧屬).
- Superclass “Angiospermae” 被子植物超綱, Class Magnoliopsida 木蘭植物綱, Subclass Magnoliidae 木蘭植物亞綱 (≡ angiosperms in Chase and Reveal 2009; APG IV 2016)
  - Amborellanae 無油樟超目
    - A1. Amborellales 無油樟目
  - B. Nymphaeanae 睡蓮超目
    - B2. Nymphaeales 睡蓮目
      - B2.3. Cabombaceae 蓴科 (≡ IIBa.36; 1/2)
        - Adding *Cabomba* 穗蓴屬 (Yang et al. 2001).
      - B2.4. Nymphaeaceae 睡蓮科 (≡ IIBa.35)
  - C. Austrobaileyanae 木蘭藤 (昆士蘭樟) 超目
    - C3. Austrobaileyales 木蘭藤 (昆士蘭樟) 目
      - C3.7. Schisandraceae 五味子科 (IIBa.25; 2/3)
        - Including IIBa.26 Illiciaceae (*Illicium*八角屬).
  - D. Magnolianae 木蘭超目
    - D4. Canellales 白樟 (白桂皮) 目
    - D5. Piperales 胡椒目
      - D5.10. Saururaceae 三白草科 (≡ IIBa.39)
      - D5.11. Piperaceae 胡椒科 (≡ IIBa.40)
      - D5.12. Aristolochiaceae 馬兜鈴科 (≡ IIBa.42)
    - D6. Magnoliales 木蘭目
      - D6.13. Myristicaceae 肉荳蔻科 (≡ IIBa.24)
      - D6.14. Magnoliaceae 木蘭科 (≡ IIBa.22)
      - D6.18. Annonaceae 番荔枝科 (≡ IIBa.23)
    - D7. Laurales 樟目
      - D7.23. Hernandiaceae 蓮葉桐科 (≡ IIBa.28)
      - D7.25. Lauraceae 樟科 (≡ IIBa.27)
  - E. Chloranthanae 金粟蘭超目
    - E8. Chloranthales 金粟蘭目
      - E8.26. Chloranthaceae 金粟蘭科 (≡ IIBa.41)
  - F. Lilianae 百合超目 (Trias-Blasi et al. 2015)
    - F9. Acorales 菖蒲目
      - F9.27. **Acoraceae** 菖蒲科 (1)
        - *Acorus* 菖蒲屬 (IIBb.29 Araceae).
    - F10. Alismatales 澤瀉目
      - F10.28. Araceae 天南星科 (IIBb.29; 16/20)
        - Adding *Syngonium* 合果芋屬 (Wu et al. 2010); excluding F9.27 Acoraceae (*Acorus*); including IIBb.30 Lemnaceae [*Landoltia* (≡ *Spirodela punctata*) 蘭氏萍屬 (Les and Crawford 1999), *Lemna* 青萍屬, *Spirodela* 浮萍屬, and *Wolffia* 無根萍屬).
      - F10.30. Alismataceae 澤瀉科 (IIBb.1; 3/5)
        - Including Limnocharitaceae (*Hydrocleys* 水罌粟屬 and *Limnocharis* 黃花藺屬; Yang et al. 2001).
        - F10.32. Hydrocharitaceae 水鱉科 (IIBb.2; 7/10)
          - Adding *Egeria* 水蘊草屬 (Wu et al. 2010) and *Limnobium* 南美海綿屬 (Wu et al. 2010); incluidng IIBb.8 Najadaceae (*Najas* 拂尾藻屬).
        - F10.34. Aponogetonaceae 水蕹科 (≡ IIBb.3)
        - F10.37. Zosteraceae 甘藻科 (≡ IIBb.7)
        - F10.38. Potamogetonaceae 眼子菜科 (IIBb.4; 1/3)
          - Adding *Stuckenia* (≡ *Potamogeton pectinatus*) 篦齒眼子菜屬 (Lindqvist et al. 2006); including IIBb.6 Zannichelliaceae *pro parte* (*Zannichellia* 角果藻屬).
        - F10.40. Ruppiaceae 流蘇菜科 (≡ IIBb.5)
        - F10.41. **Cymodoceaceae** 絲粉藻科 (3)
          - IIBb.4 Potamogetonaceae *pro parte* (*Cymodocea* 絲粉藻屬 and *Syringodium* 針葉藻屬; Lin et al. 2005) and IIBb.6 Zannichelliaceae *pro parte* (*Halodule* 二葯藻屬). Based on Ko (2004), the photographs of Lin et al. (2005; Fig. 5a, b) identified as *Thalassodendron ciliatum* are likely misidentification of *Cymodocea serrulata*.
  - F11. Petrosaviales 櫻井草 (無葉蓮) 目
    - F11.42. Petrosaviaceae 櫻井草 (無葉蓮) 科 (≡ IIBb.9A)
  - F12. Dioscoreales 薯蕷目
    - F12.43. **Nartheciaceae 沼金花 (納茜菜) 科 (1)**
      - *Aletris* 粉條兒屬 (IIBb.9 Liliaceae)
    - F12.44. Burmanniaceae 水玉簪科 (≡ IIBb.18)
    - F12.45. Dioscoreaceae 薯蕷科 (IIBb.14; 1/2)
      - Including IIBb.28(A) Taccaceae (*Tacca* 蒟蒻薯屬).
  - F13. Pandanales 露兜樹目
    - F13.46. Triuridaceae 霉草科 (≡ IIBb.39)
    - F13.48. Stemonaceae 百部科 (≡ IIBb.10)
    - F13.49. Cyclanthaceae 巴拿馬草科 (≡ IIBb.28)
    - F13.50. Pandanaceae 露兜樹科 (≡ IIBb.31)
  - F14. Liliales 百合目
    - F14.53. **Melanthiaceae** 黑葯花科 (5)
      - *Ypsilandra* (丫蕊花屬; Hsu et al. 2011) and IIBb.9 Liliaceae *pro. parte* (*Helonias* 胡麻花屬, *Paris* 七葉一枝花屬, *Trillium* 延齡草屬, and *Veratrum* 藜蘆屬).
    *F14.55. Alstroemeriaceae 百合水仙 (六出花) 科
    - F14.56. **Colchicaceae** 秋水仙科 (1)
      - *Disporum* 寶鐸花屬 (IIBb.9 Liliaceae).
    - F14.59. Smilacaceae 菝葜科 (≡ IIBb.15)
    - F14.60. Liliaceae 百合科 (IIBb.9; 21/2)
      - *Lilium* (百合屬) and *Tricyrtis* (油點草屬); excluding F14.53 Melanthiaceae (*Helonias*, *Paris*, *Trillium*, and *Veratrum*), F14.56 Colchicaceae (*Disporum*), F15.72 Asphodelaceae (*Dianella* and *Hemerocallis*), F15.73 Amaryllidaceae *pro parte* (*Allium*), and F15.74 Asparagaceae *pro parte* [*Asparagus*, *Aspidistra*, *Disporopsis*, *Liriope*, *Ophiopogon*, *Peliosanthes*, *Polygonatum*, *Rohdea* (≡ *Campylandra*), *Scilla* (≡ *Barnardia*), *Smilacina* (≡ *Maianthemum*), and *Thysanotus*].
  - F15. Asparagales 天門冬目
    - F15.61. Orchidaceae 蘭科 (≡ IIBb.38)
    - F15.66. Hypoxidaceae 仙茅科 (≡ IIBb.13)
    - F15.70. Iridaceae 鳶尾科 (≡ IIBb.17)
    - F15.72. **Asphodelaceae** 阿福花 (獨尾草) 科 (2)
      - IIBb.9 Liliaceae *pro parte* (*Dianella* 桔梗蘭屬and *Hemerocallis* 萱草屬).
    - F15.73. Amaryllidaceae 石蒜科 (IIBb.12; 2/3)
      - Including *Allium* 蔥屬 (IIBb.9 Liliaceae).
    - F15.74. **Asparagaceae** 天門冬科 (16)
      - Including IIBb.11 Agavaceae (*Agave* 龍舌蘭屬, *Cordyline* 朱蕉屬, *Dracaena* 龍血樹屬, and *Yucca* 金棒蘭屬) and IIBb.9 Liliaceae *pro. parte* [*Asparagus* 天門冬屬, *Aspidistra* 蜘蛛抱蛋屬, *Barnardia* (≡ *Scilla sinensis*) 綿棗兒屬, *Disporopsis* 假寶鐸花屬, *Heteropolygonatum* 異黃精屬 (Chao et al. 2013), *Liriope* 麥門冬屬, *Maianthemum* 鹿藥屬 (舞鶴草) 屬, *Ophiopogon* 沿階草屬, *Peliosanthes* 球子草屬, *Polygonatum* 黃精屬, *Rohdea* (≡ *Campylandra*) 萬年青屬 (Yamashita and Tamura 2004), and *Thysanotus* 異蕊草屬].
  - F16. Arecales 棕櫚目
    - F16.76. Arecaceae (≡ Palmae) 棕櫚科 (≡ IIBb.27)
  - F17. Commelinales 鴨跖草目
    - F17.78. Commelinaceae 鴨跖草科 (≡ IIBb.21)
    - F17.79. Philydraceae 田蔥科 (≡ IIBb.19)
    - F17.80. Pontederiaceae 雨久花科 (≡ IIBb.16)
  - F18. Zingiberales 薑目
    *F18.82. Strelitziaceae 旅人蕉科
    *F18.84. Heliconiaceae 蠍尾蕉 (赫蕉) 科
    - F18.85. Musaceae 芭蕉科 (≡ IIBb.34)
    - F18.86. Cannaceae 美人蕉科 (≡ IIBb.36)
    - F18.87. Marantaceae 竹芋科 (≡ IIBb.37)
    - F18.88. **Costaceae** 閉鞘薑科 (1)
      - *Costus* 閉鞘薑屬 (IIBb.35 Zingiberaceae).
    - F18.89. Zingiberaceae 薑科 (IIBb.35; 5/5)
      - Adding *Curcuma* 薑黃屬 (Wu et al. 2010); excluding F18.88. Costaceae (*Costus*).
  - F19. Poales 禾本目
    - F19.90. Typhaceae 香蒲科 (IIBb.33; 1/2)
      - Including IIBb.33 Sparganiaceae (*Sparganium* 黑三稜屬).
    *F19.91. Bromeliaceae 鳳梨科
    - F19.93. Xyridaceae 蔥草科 (≡ IIBb.22)
    - F19.94. Eriocaulaceae 穀精草科 (≡ IIBb.23)
    - F19.97. Juncaceae 燈心草科 (≡ IIBb.20)
    - F19.98. Cyperaceae 莎草科 (≡ IIBb.25)
    - F19.100. Flagellariaceae 鞭藤科 (≡ IIBb.24)
    - F19.103. Poaceae (≡ Gramineae) 禾本科 (≡ IIBb.26)
  - G. Ceratophyllanae 金魚藻超目
    - G20. Ceratophyllales 金魚藻目
      - G20.104. Ceratophyllaceae 金魚藻科 (≡ IIBa.38)
  - H. Ranunculanae 毛茛超目
    - H21. Ranunculales 毛茛目
      - H21.106. Papaveraceae 罌粟科 (IIBa.48; 3/5)
        - Including IIBa.48A Fumariaceae (*Corydalis* 紫蓳屬 and *Fumaria* 球果紫菫屬).
      - H21.108. Lardizabalaceae 木通科 (≡ IIBa.32)
      - H21.109. Menispermaceae 防己科 (≡ IIBa.33)
      - H21.110. Berberidaceae 小檗科 (≡ IIBa.31)
      - H21.111. Ranunculaceae 毛茛科 (≡ IIBa.30)
  - I. Proteanae 山龍眼超目
    - I22. Proteales 山龍眼目
      - I22.112. Sabiaceae 清風藤科 (≡ IIBa.72)
      - I22.113. Nelumbonaceae 蓮科 (≡ IIBa.34)
      - I22.115. Proteaceae 山龍眼科 (≡ IIBa.9)
  - J. Trochodendranae 昆欄樹超目
    - J23. Trochodendrales 昆欄樹目
      - J23.116. Trochodendraceae 昆欄樹科 (≡ IIBa.29)
  - K. Buxanae 黃楊超目
    - K24. Buxales 黃楊目
      - K24.117. Buxaceae 黃楊科 (≡ IIBa.77)
  - L. Myrothamnanae 摺扇葉超目
    - L25. Gunnerales 大葉草 (洋二仙草) 目
  - M. Dillenianae 第倫桃超目
    - M26. Dilleniales 第倫桃目
      *M26.120. Dilleniaceae 第倫桃科
  - N. Saxifraganae 虎耳草超目
    - N27. Saxifragales 虎耳草目
      - N27.123. **Altingiaceae** 蕈樹 (楓香) 科 (1)
        - *Liquidambar* 楓香屬 (IIBa.51 Hamamelidaceae).
      - N27.124. Hamamelidaceae 金縷梅科 (IIBa.51; 6/5)
        - Excluding *Liquidambar* (N27.123).
      - N27.126. Daphniphyllaceae 虎皮楠科 (≡ IIBa.62)
      - N27.127. **Iteaceae** 鼠刺科 (1)
        - *Itea* 鼠刺屬 (IIBa.53 Saxifragaceae).
      - N27.128. **Grossulariaceae** 茶藨子科 (1)
        - *Ribes* 茶藨子屬 (IIBa.53 Saxifragaceae).
      - N27.129. Saxifragaceae 虎耳草科 (IIBa.53; 13/5)
        - *Astilbe* 落新婦屬, *Chrysosplenium* 貓兒眼睛草屬, *Mitella* 嗩吶草屬, *Saxifraga* 虎耳草屬, and *Tiarella* 黃水枝屬; excluding S48.320 Hydrangeaceae (*Cardiandra*, *Deutzia*, *Hydrangea*, *Pileostegia*, and *Schizophragma*), N27.127 Iteaceae (*Itea*), N27.128 Grossulariaceae (*Ribes*), and *Parnassia* (O34.168).
      - N27.130. Crassulaceae 景天科 (≡ IIBa.52)
      - N27.134. Haloragaceae 小二仙草科 (≡ IIBa.102)
  - O. Rosanae 薔薇超目
    - O28. Vitales 葡萄目
      - O28.136. Vitaceae 葡萄科 (IIBa.80; 6/7)
        - Including IIBa.81 Leeaceae (*Leea* 火筒樹屬).
    - O29. Zygophyllales 蒺藜目
      - O29.138. Zygophyllaceae 蒺藜科 (≡ IIBa.60)
    - O30. Fabales 豆目
      - O30.140. Fabaceae (≡ Leguminosae) 豆科 (≡ IIBa.57)
      - O30.141. **Surianaceae** 海人樹科 (1)
        - *Suriana* 海人樹屬 (Huang et al. 1994).
        - O30.142. Polygalaceae 遠志科 (≡ IIBa.67)
    - O31. Rosales 薔薇目
      - O31.143. Rosaceae 薔薇科 (≡ IIBa.55)
      - O31.146. Elaeagnaceae 胡頹子科 (≡ IIBa.88)
      - O31.147. Rhamnaceae 鼠李科 (≡ IIBa.79)
      - O31.148. Ulmaceae 榆科 (IIBa.6; 5/2)
        - *Ulmus* 榆屬 and *Zelkova* 櫸屬; excluding O31.149 Cannabaceae *pro parte* (*Aphananthe*, *Celtis*, and *Trema*).
      - O31.149. **Cannabaceae** 大麻科 (4)
        - *Humulus* 葎草屬 (IIBa.7 Moraceae) and IIBa.6 Ulmaceae *pro parte* (*Aphananthe* 糙葉樹屬, *Celtis* 朴屬, and *Trema* 山黃麻屬).
      - O31.150. Moraceae 桑科 (IIBa.7; 8/7)
        - Excluding *Hummulus* (O31.149).
      - O31.151. Urticaceae 蕁麻科 (IIBa.8; 21/22)
        - Including IIBa.7A Cecropiaceae (*Poikilospermum* 錐頭麻屬).
    - O32. Fagales 殼斗目
      - O32.153. Fagaceae 殼斗科 (≡ IIBa.5)
      - O32.154. Myricaceae 楊梅科 (≡ IIBa.1)
        - *Morella* (= *Myrica*) 楊梅屬 (Herbert 2005; Huguet et al. 2005).
        - O32.155. Juglandaceae 胡桃科 (≡ IIBa.2)
        *O32.156. Casuarinaceae 木麻黃科
        - O32.158. Betulaceae 樺木科 (≡ IIBa.4)
    - O33. Cucurbitales 葫蘆 (瓜) 目
      - O33.162. Coriariaceae 馬桑科 (≡ IIBa.68)
      - O33.163. Cucurbitaceae 葫蘆 (瓜) 科 (≡ IIBa.94)
      - O33.166. Begoniaceae 秋海棠科 (≡ IIBa.93)
    - O34. Celastrales 衛矛目
      - O34.168. Celastraceae 衛矛科 (IIBa.75; 6/6)
      - Including *Parnassia* 梅花草屬 (IIBa.53 Saxifragaceae); excluding *Perrottetia* (O41.234).
    - O35. Oxalidales 酢漿草目
      - O35.170. Connaraceae 牛栓藤科 (≡ IIBa.56)
      - O35.171. Oxalidaceae 酢漿草科 (≡ IIBa.58)
      - O35.173. Elaeocarpaceae 杜英科 (≡ IIBa.82)
    - O36. Malpighiales 黃褥花 (金虎尾) 目
      - O36.179. Rhizophoraceae 紅樹科 (≡ IIBa.99)
      *O36.181. Ochnaceae 金蓮木科
      - O36.183. Clusiaceae (≡ Guttiferae) 藤黃科 (IIBa.46; 4/1)
        - *Garcinia* 福木屬; excluding O36.184 Calophyllaceae (*Calophyllum*) and O36.186 Hypericaceae (*Hypericum* and *Triadenum*).
        - O36.184. **Calophyllaceae** 胡桐 (紅厚殼) 科 (1)
          - *Calophyllum* 胡桐屬 (IIBa.46 Guttiferae).
        - O36.186. **Hypericaceae** 金絲桃科 (2)
          - IIBa.46 Guttiferae *pro parte* (*Hypericum* 金絲桃屬 and *Triadenum*三腺金絲桃屬).
        - O36.189. **Putranjivaceae** 非洲核果木 (核果木) 科 (1)
          - *Drypetes* (including *Liodendron*) 鐵色屬 (IIBa.61 Euphorbiaceae).
        - O36.191. Elatinaceae 溝繁縷科 (≡ IIBa.92)
        - O36.192. Malpighiaceae 黃褥花 (金虎尾) 科 (≡ IIBa.66)
        *O36.197. Chrysobalanaceae 可可李 (金殼果) 科
        - O36.200. Violaceae 堇菜科 (≡ IIBa.90)
        - O36.202. Passifloraceae 西番蓮科 (≡ IIBa.91A)
        - O36.204. Salicaceae 楊柳科 (IIBa.3) (1/7)
          - *Salix* 柳屬; including IIBa.89 Flacourtiaceae (*Casearia* 嘉賜木屬, *Flacourtia* 羅庚果屬, *Homalium* 天料木屬, *Idesia* 山桐子屬, *Scolopia* 魯花樹屬, and *Xylosma* 柞木屬).
        - O36.207. Euphorbiaceae 大戟科 (IIBa.61; 27/17)
          - *Acalypha* 鐵莧屬, *Alchornea* 山麻桿屬, *Claoxylon* 假鐵莧屬, *Croton* 巴豆屬, *Euphorbia* (including *Chamaesyce*) 大戟屬, *Excoecaria* 土沉香屬, *Homonoia* 水楊梅屬, *Macaranga* 血桐屬, *Mallotus* 野桐屬, *Melanolepis* 蟲屎屬, *Mercurialis* 山靛屬, *Homalanthus* (≡ *Omalanthus*) 圓葉血桐屬, *Ricinus* 蓖麻屬, and *Sapium* 烏桕屬, and *Suregada* (including *Gelonium*) 白樹屬; adding *Manihot* 木薯屬 and *Vernicia* (including *Aleurites montana*; Wu et al. 2010) 油桐屬; excluding O36.189 Putranjivaceae (*Drypetes* and *Liodendron*) and O36.211 Phyllanthaceae (*Antidesma*, *Bischofia*, *Breynia*, *Bridelia*, *Flueggea*, *Glochidion*, *Margaritaria*, *Phyllanthus*, and *Synostemon*).
        - O36.208. **Linaceae** 亞麻科 (1)
          - *Linum* 亞麻屬 (Chao et al. 2017).
        - O36.211. **Phyllanthaceae** 葉下珠科 (8)
          - IIBa.61 Euphorbiaceae *pro parte* (*Antidesma* 五月茶屬, *Bischofia* 重陽木屬, *Breynia* (including *Synostemon*) 山漆莖屬, *Bridelia* 土密樹屬, *Flueggea* 白飯樹屬, *Glochidion* 饅頭果屬, *Margaritaria* 紫黃屬, and *Phyllanthus* 油柑屬).
    - O37. Geraniales 牻牛兒苗目
      - O37.212. Geraniaceae 牻牛兒苗科 (≡ IIBa.59)
    - O38. Myrtales 桃金孃目
      - O38.214. Combretaceae 使君子科 (≡ IIBa.100)
      - O38.215. Lythraceae 千屈菜科 (IIBa.95; 5/6)
        - Including IIBa.101A Trapaceae (*Trapa* 菱屬) and *Punicaceae.
      - O38.216. Onagraceae 柳葉菜科 (≡ IIBa.101)
      - O38.218. Myrtaceae 桃金孃科 (≡ IIBa.96)
      - O38.219. Melastomataceae 野牡丹科 (≡ IIBa.98)
    - O39. Crossosomatales 流蘇子 (纓子木) 目
      - O39.226. Staphyleaceae 省沽油科 (≡ IIBa.76)
      - O39.228. Stachyuraceae 旌節花科 (≡ IIBa.91)
    - O40. Picramniales 美洲苦木目
    - O41. Huerteales 腺椒樹 (十齒花) 目
      - O41.234. **Dipentodontaceae** 十齒花科 (1)
        - *Perrottetia* 核子木屬 (IIBa.75 Celastraceae).
    - O42. Sapindales 無患子目
      *O42.238. Burseraceae 橄欖科
      - O42.239. Anacardiaceae 漆樹科 (≡ IIBa.69)
      - O42.240. Sapindaceae 無患子科 (IIBa.71; 9/10)
        - Including IIBa.70 Aceraceae (*Acer* 楓屬).
      - O42.241. Rutaceae 芸香科 (≡ IIBa.63)
      - O42.242. Simaroubaceae 苦木科 (≡ IIBa.64)
      - O42.243. Meliaceae 楝科 (≡ IIBa.65)
    - O43. Malvales 錦葵目
      - O43.245. **Muntingiaceae** 文定果 (西印度櫻桃) 科 (1)
        - *Muntingia* 西印度櫻桃屬 (IIBa.83 Tiliaceae).
      - O43.247. Malvaceae 錦葵科 (IIBa.84; 8/26)
        - *Abelmoschus* 秋葵屬, *Abutilon* 莔麻屬, *Hibiscus* 木槿屬, *Malachra* 玄葵屬, *Malva* 錦葵屬, *Malvastrum* 賽葵屬, *Sida* 金午時花屬, *Thespesia* 繖楊屬, and *Urena* 野棉花屬; adding *Anoda* 蔓錦葵屬 (Li and Wang 2012) and *Modiola* 蔓葵屬 (Wu et al. 2010); including IIBa.85 Bombacaceae (*Bombax* 木棉屬 and *Pachira* 馬拉巴栗屬; Wu et al. 2010), IIBa.86 Sterculiaceae (*Firmiana* 梧桐屬, *Helicteres* 山芝麻屬, *Heritiera* 銀葉樹屬, *Kleinhovia* 克蘭樹屬, *Melochia* 野路葵屬, *Pterospermum* 翅子樹屬, *Reevesia* 梭羅樹屬, *Sterculia* 蘋婆屬, and *Waltheria* 草梧桐屬) and IIBa.83 Tiliaceae *pro parte* (*Berrya* 六翅木屬, *Corchorus* 黃麻屬, *Grewia* 捕魚木屬, and *Triumfetta* 垂桉草屬).
      - O43.249. Thymelaeaceae 瑞香科 (≡ IIBa.87)
      *O43.250. Bixaceae 胭脂樹 (紅木) 科
      *O43.253. Dipterocarpaceae 龍腦香科
    - O44. Brassicales 十字花目
      - O44.254. **Akaniaceae** 疊珠樹科 (1)
        - Including IIBa.71A Bretschneideraceae (*Bretschneidera* 鐘萼木屬).
      *O44.255. Tropaeolaceae 金蓮花 (旱金蓮) 科
      - O44.257. **Caricaceae** 番木瓜科 (1)
        - *Carica* 番木瓜屬 (Wu et al. 2010).
      - O44.268. Capparaceae 山柑科 (IIBa.49; 3/2)
        - Excluding *Cleome* (O44.269).
      - O44.269. **Cleomaceae** 白花菜 (醉蝶花) 科 (1)
        - *Cleome* 白花菜屬 (IIBa.49 Capparaceae).
      - O44.270. Brassicaceae (≡ Cruciferae) 十字花科 (≡ IIBa.50)
  - P. Berberidopsidanae 紅珊藤 (智利藤) 超目
    - P45. Berberidopsidales 紅珊藤 (智利藤) 目
  - Q. Santalanae 檀香超目
    - Q46. Santalales 檀香目
      - Q46.273. Olacaceae 鐵青樹科 (IIBa.9A; 2/1)
        - Excluding *Schoepfia* (Q46.278).
      - Q46.274. Opiliaceae 山柚科 (≡ IIBa.10A)
      - Q46.275. Balanophoraceae 蛇菰科 (≡ IIBa.12)
      - Q46.276. Santalaceae 檀香科 (IIBa.10; 1/3)
        - *Thesium* (百蕊草屬); including IIBa.11 Loranthaceae *pro parte* (*Korthalsella* 檜葉寄生屬and *Viscum* 槲寄生屬).
      - Q46.278. **Schoepfiaceae** 青皮木科 (1)
        - *Schoepfia* 青皮木屬 (IIBa.9A Olacaceae)
      - Q46.279. Loranthaceae 桑寄生科 (IIBa.11; 4/2)
        - *Loranthus* 桑寄生屬 and *Taxillus* 鈍果桑寄生屬; excluding Q46.276 Santalaceae *pro parte* (*Korthalsella* and *Viscum*).
  - R. Caryophyllanae 石竹超目
    - R47. Caryophyllales 石竹目
      - R47.281. **Tamaricaceae** 檉柳科 (1)
        - *Tamarix* 檉柳屬 (Wu et al. 2010).
      - R47.282. Plumbaginaceae 藍雪科 (≡ IIBa.113)
      - R47.283. Polygonaceae 蓼科 (≡ IIBa.13)
      - R47.284. Droseraceae 茅膏菜科 (≡ IIBa.47)
      - R47.295. Caryophyllaceae 石竹科 (≡ IIBa.19)
      - R47.297. Amaranthaceae 莧科 (IIBa.21; 9/14)
        - Adding Digera 瘤果莧 (Wang and Chen 2013) and Pupalia 鉤牛膝屬 (Wu et al.^ 2010^); including IIBa.20 Chenopodiaceae (*Atriplex* 濱藜屬, *Chenopodium* 藜屬, and *Suaeda* 鹼蓬屬).
      - R47.304. Aizoaceae 番杏科 (≡ IIBa.17)
      - R47.305. Phytolaccaceae 商陸科 (IIBa.14; 2/1) (Wu et al.  2010)
        - Excluding *Rivina* (R47.306).
      - R47.306. **Petiveriaceae** 蒜香草科 (1)
        - *Rivina* 珊瑚珠屬 (IIBa.14 Phytolaccaceae; Wu et al. 2010).
      - R47.308. Nyctaginaceae 紫茉莉科 (≡ IIBa.15)
      - R47.309. Molluginaceae 粟米草科 (≡ IIBa.16)
      *R47.311. Didiereaceae 刺戟木 (龍樹) 科
      - R47.312. Basellaceae 落葵科 (≡ IIBa.18A)
      - R47.314. **Talinaceae** 土人參科 (1)
        - *Talinum* 土人參屬 (IIBa.18 Portulacaceae).
      - R47.315. Portulacaceae 馬齒莧科 (IIBa.18; 2/1)
        - Excluding *Talinum* (R47.314).
      - R47.317. **Cactaceae** 仙人掌科 (4)
        - *Cereus* 六角柱屬, *Epiphyllum* 曇花屬, *Hylocereus* 量天尺屬, and *Opuntia* 仙人掌屬 (Wu et al. 2010).
  - S. Asteranae 菊超目
    - S48. Cornales 山茱萸目
    *S48.318. Nyssaceae 珙桐科
    - S48.320. **Hydrangeaceae** 八仙花 (繡球) 科 (2)
      - IIBa.53 Saxifragaceae *pro parte* [*Deutzia* 溲疏屬and *Hydrangea* (including *Cardiandra*, *Pileostegia*, and *Schizophragma*) 八仙花屬 (De Smet et al. 2015)].
      - S48.324. Cornaceae 山茱萸科 (IIBa.105: 4/2)
        - *Cornus* (including *Benthamidia* and *Swida*) 山茱萸屬; including IIBa.104 Alangiaceae (*Alangium* 八角楓屬); excluding *Aucuba* (S52.351) and *Helwingia* (S58.391).
    - S49. Ericales 杜鵑花目
      - S49.325. Balsaminaceae 鳳仙花科 (≡ IIBa.73)
      *S49.329. Polemoniaceae 花荵 (花蔥) 科
      - S49.330. Lecythidaceae 玉蕊科 (≡ IIBa.97)
      - S49.332. **Pentaphylacaceae** 五列木科 (5)
        - IIBa.45 Theaceae *pro parte* (*Adinandra* 楊桐屬, *Anneslea* 茶梨屬, *Cleyera* 紅淡比屬, *Eurya* 柃木屬, and *Ternstroemia* 厚皮香屬).
      - S49.333. Sapotaceae 山欖科 (≡ IIBa.114)
      - S49.334. Ebenaceae 柿樹科 (≡ IIBa.115)
      - S49.335. Primulaceae 報春花科 (IIBa.112; 5/8)
        - *Androsace* 點地梅屬, *Lysimachia* (including *Anagalis*; Manns and Anderberg 2009) 珍珠菜屬, *Primula* 櫻草屬, and *Stimpsonia* 施丁草屬; including IIBa.111 Myrsinaceae (*Ardisia* 紫金牛屬, *Embelia* 藤木槲屬, *Maesa* 山桂花屬, and *Myrsine* 竹杞屬).
      - S49.336. Theaceae 茶科 (IIBa.45; 9/4)
        - *Camellia* 山茶屬, *Gordonia* 大頭茶屬, *Pyrenaria* 烏皮茶屬, and *Schima* 木荷屬; excluding S49.332 Pentaphylacaceae (*Adinandra*, *Anneslea*, *Cleyera*, *Eurya*, and *Ternstroemia*)
      - S49.337. Symplocaceae 灰木科 (≡ IIBa.117)
      - S49.338. Diapensiaceae 岩梅科 (≡ IIBa.108)
      - S49.339. Styracaceae 安息香科 (≡ IIBa.116)
      - S49.342. Actinidiaceae 獼猴桃科 (≡ IIBa.44)
      - S49.345. **Mitrastemonaceae** 奴草 (帽蕊草) 科 (1)
        - *Mitrastemon* 奴草屬 (IIBa.43: Rafflesiaceae).
    - S49.346. Ericaceae 杜鵑花科 (IIBa.110; 6/11)
      - Including IIBa.109 Pyrolaceae (*Cheilotheca* 水晶蘭屬, *Chimaphila* 愛冬葉屬, *Moneses* 單花鹿蹄草屬, *Monotropa* 錫杖花屬, and *Pyrola* 鹿蹄草屬).
      - S50. Icacinales 茶茱萸目
        - S50.348. Icacinaceae 茶茱萸科 (IIBa.78; 3/1)
          - *Nothapodytes* 鷹紫花樹屬; excluding *Gomphandra* (S58.388) and *Gonocaryum* (S58.389).
      - S51. Metteniusales 水螅花目
      - S52. Garryales 絲纓花目
        *S52.350. Eucommiaceae 杜仲科
        - S52.351. **Garryaceae** 絲纓花科 (1)
          - *Aucuba* 桃葉珊瑚屬 (IIBa.105 Cornaceae).
      - S53. Gentianales 龍膽目
        - S53.352. Rubiaceae 茜草科 (IIBa.123; 38/46)
          - *Argostemma* 水冠草屬, *Canthium* 朴萊木屬, *Cephalanthus* 風箱樹屬, *Coptosapelta* 瓢簞藤屬, *Damnacanthus* 伏牛花屬, *Dentella* 小牙草屬, *Diodia* 鈕扣草屬, *Galium* 豬殃殃屬, *Gardenia* 黃槴屬, *Geophila* 苞花蔓屬, *Guettarda* 葛塔德木屬, *Hedyotis* 耳草屬, *Ixora* 仙丹花屬, *Knoxia* 諾氏草屬, *Lasianthus* 雞屎樹屬, *Litosanthes* 壺冠木屬, *Mitchella* 蔓虎刺屬, *Mitracarpus* 蓋裂果屬 (Ling and Chen 2013), *Morinda* 羊角藤屬, *Mussaenda* 玉葉金花屬, *Neanotis* 新耳草屬, *Neonauclea* 欖仁舅屬, *Nertera* 深柱夢草屬, *Oldenlandiopsis*微耳草屬 (Jung et al. 2011), *Ophiorrhiza* 蛇根草屬 (including *Hayataella*; Nakamura et al. 2006), *Paederia* 雞屎藤屬, *Pavetta* 茜木屬, *Psychotria* 九節木屬, *Randia* 茜草樹屬, *Richardia* 擬鴨舌癀屬, *Rubia* 茜草屬, *Serissa* 滿天星屬 (Wu et al. 2010), *Sherardia* 雪亞迪草屬 (Wu et al. 2010), *Sinoadina* 水冬瓜屬, *Spermacoce* 擬鴨舌癀舅屬 (includng *Hemidiodia* in Wu et al. 2010), *Tarenna* 玉心花屬, *Timonius* 貝木屬, *Tricalysia* 狗骨仔屬, *Uncaria* 鉤藤屬, and *Wendlandia* 水錦樹屬; including IIBa.103 Theligonaceae (*Theligonum* 纖花草屬). Based on Neupane et al. (2015), the genera *Dimetia* 涼喉茶屬 (新擬), *Exallage* 金毛耳草屬 (新擬), *Leptopetalum* 脈耳草屬 (新擬), *Oldenlandia* 龍吐珠屬 (新擬), and *Scleromitrion* 蛇舌草屬 are segregated from the Taiwanese species of *Hedyotis* (Hsu and Chen 2017).
        - S53.353. Gentianaceae 龍膽科 (IIBa.120; 7/7)
          - Including *Fagraea* 灰莉屬 (IIBa.119 Loganiaceae); excluding *Nymphoides* (S59.400).
        - S53.354. Loganiaceae 馬錢科 (IIBa.119; 6/4)
          - Excluding *Fagraea* (S53.353) and *Buddleja* (S57.371).
        - S53.356. Apocynaceae 夾竹桃科 (IIBa.121; 10/24)
          - *Alyxia* 念珠藤屬, *Anodendron* 錦蘭屬, *Cerbera*海檬果屬, *Holarrhena* 止瀉木屬, *Melodinus* 山橙屬, *Parsonsia* 爬森藤屬, *Rauvolfia* 蘿芙木屬, *Tabernaemontana* 馬蹄花屬, *Trachelospermum*絡石屬, *Urceola* (including *Ecdysanthera*) 水壺藤屬; adding *Alstonia* 黑板樹屬 (Wu et al. 2010) and *Catharanthus* 長春花屬 (*Vinca* in Wu et al. 2010); including IIBa.122 Asclepiadaceae (*Asclepias* 尖尾鳳屬, *Cryptolepis* 隱鱗藤屬, *Cynanchum* 牛皮消屬, *Dischidia* 風不動屬, *Dregea* 華他卡藤屬, *Gymnema* 武靴藤屬, *Heterostemma* 布朗藤屬, *Hoya* 毬蘭屬, *Jasminanthes* 舌瓣花屬, *Marsdenia* 牛彌菜屬, *Telosma* 夜香花屬, and *Tylophora* 鷗蔓屬).
      - S54. Boraginales 紫草目 (sensu Luebert et al.^ 2016^)
        - S54.357. Boraginaceae 紫草科 (IIBa.126; 12/8)
          - *Bothriospermum* 細纍子草屬, *Cynoglossum* 琉璃草屬, *Lithospermum* 紫草屬, *Thyrocarpus* 盾果草屬, *Trichodesma* 碧果草屬, and *Trigonotis* 附地草屬; adding *Myosotis* 勿忘草屬 (Wu et al. 2010) and *Symphytum* 聚合草屬 (Wu et al. 2010); excluding S54.357B Coldeniaceae (*Coldenia*), S54.357C Cordiaceae (*Cordia*), S54.357D Ehretiaceae (*Carmona* and *Ehretia*), and S54.357E Heliotropiaceae (*Heliotropium* and *Tournefortia*).
          - S54.357B. **Coldeniaceae** 生果草科 (1)
            - *Coldenia* 生果草屬 (IIBa.126 Boraginaceae).
          - S54.357C. **Cordiaceae** 破布子科 (1)
            - *Cordia* 破布子屬 (IIBa.126 Boraginaceae).
          - S54.357D. **Ehretiaceae** 厚殼樹科 (1)
            - *Ehretia* (including *Carmona*) 厚殼樹屬 (IIBa.126 Boraginaceae).
          - S54.357E. **Heliotropiaceae** 天芹菜科 (1)
            - *Heliotropium* (including *Tournefortia*) 天芹菜屬 (IIBa.126 Boraginaceae).
      - S55. Vahliales 黃漆姑目
      - S56. Solanales 茄目
        - S56.359. Convolvulaceae 旋花科 (≡ IIBa.124)
        - S56.360. Solanaceae 茄科 (≡ IIBa.130)
        - S56.362. Sphenocleaceae 密穗桔梗 (尖瓣花) 科 (≡ IIBa.142A)
        - S56.363. **Hydroleaceae** 田基麻科 (1)
          - *Hydrolea* 探芹草屬 (IIBa.125 Hydrophyllaceae)
      - S57. Lamiales 唇形目
        - S57.366. Oleaceae 木犀科 (≡ IIBa.118)
        - S57.369. Gesneriaceae 苦苣苔科 (≡ IIBa.134)
        - S57.370. Plantaginaceae 車前科 (IIBa.138; 1/16)
          - *Plantago* 車前屬; including IIBa.128 Callitrichaceae (*Callitriche* 水馬齒屬) and IIBa.131 Scrophulariaceae *pro parte* [*Antirrhinum* 金魚草屬 (Chen and Wang 2014), *Bacopa* 過長沙屬, *Deinostema* 澤番椒屬, *Digitalis* 毛地黃屬, *Dopatrium* 虻眼草屬, *Ellisiophyllum* 海螺菊屬, *Hemiphragma* 腰只花屬, *Limnophila* 石龍尾屬, *Mecardonia* 過長沙舅屬, *Microcarpaea* 微果草屬, *Scoparia* 野甘草屬, *Stemodia* (孿生花屬; Liang et al. 2011), *Veronica* 婆婆納屬, and *Veronicastrum* 腹水草屬].
        - S57.371. Scrophulariaceae 玄參科 (IIBa.131; 26/3)
          - *Scrophularia* (玄參屬); including *Buddleja* 揚波屬 (IIBa.119 Loganiaceae) and IIBa.137 Myoporaceae (*Myoporum* 苦藍盤屬); excluding S57.370 Plantaginaceae *pro parte* (*Bacopa*, *Deinostema*, *Digitalis*, *Dopatrium*, *Ellisiophyllum*, *Hemiphragma*, *Limnophila*, *Mecardonia*, *Microcarpaea*, *Scoparia*, *Veronica*, and *Veronicastrum*), S57.373 Linderniaceae (*Legazpia*, *Lindernia*, and *Torenia*), S57.384 Mazaceae (*Mazus*), S57.385 Phrymaceae (*Mimulus*), S57.386 Paulowniaceae (*Paulownia*), and S57.387 Orobanchaceae *pro parte* (*Alectra*, *Centranthera*, *Euphrasia*, *Pedicularis*, *Phtheirospermum*, *Siphonostegia*, and *Striga*).
        - S57.373. **Linderniaceae** 母草科 (4)
          - IIBa.131 Scrophulariaceae *pro parte* [*Legazpia* 三翅萼屬, *Lindernia* 母草屬, *Micranthemum* 珍珠草屬 (Hsu et al. 2016a), and *Torenia* 倒地蜈蚣屬].
        *S57.376. Pedaliaceae 胡麻 (芝麻) 科
        - S57.377. Acanthaceae 爵床科 (IIBa.133; 15/20)
          - Adding *Asystasia* 十萬錯屬 (Wu et al. 2010), *Nelsonia* 瘤子草屬 (Wang et al. 2016), and *Thunbergia* 鄧伯花屬 (Wu et al. 2010); including *Avicennia* 海茄苳屬 (IIBa.127 Verbenaceae).
        - S57.378. Bignoniaceae 紫葳科 (≡ IIBa.132)
        - S57.379. Lentibulariaceae 狸藻科 (≡ IIBa.136)
        - S57.382. Verbenaceae 馬鞭草科 (IIBa.127; 11/5)
          - *Lantana* 馬纓丹屬, *Phyla* 鴨舌癀屬, *Stachytarpheta* 木馬鞭屬, *Verbena* 馬鞭草屬; adding *Duranta* 假連翹屬 (Wu et al. 2010); excluding *Avicennia* (S57.377) and S57.383 Lamiaceae *pro parte* (*Callicarpa*, *Caryopteris*, *Clerodendrum*, *Premna*, *Sphenodesme*, and *Vitex*).
        - S57.383. Lamiaceae (≡ Labiatae) 唇形科 (IIBa.129; 36/43)
          - Including IIBa.127 Verbenaceae *pro parte* [*Callicarpa* 紫珠屬, *Caryopteris* 蕕屬, *Clerodendrum* 海州常山屬, *Premna* 魚臭木屬, *Sphenodesme* 楔翅藤屬, *Tectona* 柚木屬 (Wu et al. 2010), and *Vitex* 牡荊屬].
        - S57.384. **Mazaceae** 通泉草科 (1)
          - *Mazus* 通泉草屬 (IIBa.131 Scrophulariaceae)
        - S57.385. **Phrymaceae** 蠅毒草 (透骨草) 科 (3)
          - *Erythranthe* (≡ *Mimulus*; Barker et al. 2012) 溝酸漿屬 (IIBa.131 Scrophulariaceae), *Peplidium* 溝馬齒屬 (Hsu et al. 2016b), and *Phryma* 蠅毒草屬 (Jung et al. 2005).
        - S57.386. **Paulowniaceae** 泡桐科 (1)
          - *Paulownia* 泡桐屬 (IIBa.131 Scrophulariaceae).
        - S57.387. Orobanchaceae 列當科 (IIBa.135; 4/14)
          - *Aeginetia* 野菰屬, *Boschniakia* 草蓯容屬, *Christisonia* 假野菰屬, *Orobanche* 列當屬; adding *Phacellanthus* 黃筒花屬 (Chung et al. 2010b); including IIBa.131 Scrophulariaceae *pro parte* [*Alectra* 黑蒴屬, *Centranthera* 距蕊花屬, *Euphrasia* 碎雪草屬, *Lathraea* 齒鱗草屬 (Chung et al. 2010a), *Melampyrum*山羅花屬 (Chen and Wang 2009), *Pedicularis* 馬先蒿屬, *Phtheirospermum* 松蒿屬, *Siphonostegia* 陰行草屬, and *Striga* 獨腳金屬].
      - S58. Aquifoliales 冬青目
        - S58.388. **Stemonuraceae** 金檀木 (粗絲木) 科 (1)
          - *Gomphandra* 毛蕊木屬 (IIBa.78 Icacinaceae)
        - S58.389. **Cardiopteridaceae** 心翼果科 (1)
          - *Gonocaryum* 瓊欖屬 (IIBa.78 Icacinaceae)
        - S58.391. **Helwingiaceae** 青莢葉科 (1)
          - *Helwingia* 青莢葉屬 (IIBa.105 Cornaceae)
        - S58.392. Aquifoliaceae 冬青科 (≡ IIBa.74)
      - S59. Asterales 菊目
        - S59.394. Campanulaceae 桔梗科 (≡ IIBa.142)
        - S59.400. **Menyanthaceae** 睡菜科 (1)
          - *Nymphoides* 莕菜屬 (IIBa.120 Gentianaceae)
        - S59.401. Goodeniaceae 草海桐科 (≡ IIBa.143)
        - S59.403. Asteraceae (≡ Compositae) 菊科 (≡ IIBa.144)
      - S60. Escalloniales 南鼠刺 (吊片果) 目
      - S61. Bruniales 絨球花目
      - S62. Paracryphiales 盔被花 (盔瓣花) 目
      - S63. Dipsacales 川續斷目
        - S63.408. **Adoxaceae** 五福花科 (2)
          - IIBa.139 Caprifoliaceae *pro parte* (*Sambucus* 接骨木屬 and *Viburnum* 莢蒾屬).
        - S63.409. Caprifoliaceae 忍冬科 (IIBa.139; 4/6)
          - *Abelia* 六道木屬 and *Lonicera* 忍冬屬; including IIBa.141 Dipsacaceae (*Scabiosa* 山蘿蔔屬) and IIBa.140 Valerianaceae (*Patrinia* 敗醬屬, *Triplostegia* 雙參屬, and *Valeriana* 纈草屬); excluding S63.408 Adoxaceae (*Sambucus* and *Viburnum*).
      - S64. Apiales 繖形目
        - S64.413. Pittosporaceae海桐科 (≡ IIBa.54)
        - S64.414. Araliaceae五加科 (IIBa.106; 10/11)
          - Including *Hydrocotyle* 天胡荽屬 (IIBa.107 Umbelliferae).
        - S64.416. Apiaceae (≡ Umbelliferae) 繖形科 (IIBa.107; 18/18)
          - Adding *Foeniculum* 茴香屬 (Wu et al. 2010); excluding *Hydrocotyle* (S64.414).

## Additional file

**Additional file 1: Appendix S1.** List of seed plant genera of Taiwan and their familial classification under the Phylogenetic Classification of Seed Plants of Taiwan.

## Abbreviations

APG: Angiosperm Phylogeny Group
FOT: Flora of Taiwan
TaiBIF: Taiwan Biodiversity Information Facility
TaiCOL: Catalogue of Life in Taiwan
TaiEOL: Taiwan Encyclopedia of Life
TaiBOL: Cryobanking Program for Wildlife Genetic Material in Taiwan
TELDAP: Taiwan e-Learning and Digital Archives Programs
GBIF: Global Biodiversity Information Facility
TaiBNET: Taiwan Biodiversity National Information Network

## References

[CR1] Barker WR, Nesom GL, Beardsley PM, Fraga NS (2012). A taxonomic conspectus of Phrymaceae: a narrowed circumscription for *Mimulus*, new and resurrected genera, and new names and combinations. Phytoneuron.

[CR2] Boufford DE, Ohashi H, Huang T-C, Hsieh C-F, Tsai J-L, Yang K-C, Peng C-I, Kuoh C-S, Hsiao A (2003) A checklist of the vascular plants of Taiwan. In: Editorial Committee of the Flora of Taiwan (ed) Flora of Taiwan, vol 6, 2nd edn. Department of Botany, National Taiwan University, Taipei, pp 15–139

[CR4] Chao C-T, Tseng Y-H, Tseng H-Y (2013). *Heteropolygonatum altelobatum* (Asparagaceae), *comb. nova*. Ann Bot Fennici.

[CR3] Chao C-T, Chen P-H, Tseng H-Y (2017). *Linum usitatissimum* L. (Linaceae), a newly naturalized species in Taiwan. Quart J For Res.

[CR5] Chapman AD (2005). Principles of data quality, version 10.

[CR6] Chase MW, Reveal JL (2009). A phylogenetic classification of the land plants to accompany APG III. Bot J Lin Soc.

[CR7] Chen C-H, Wang C-M (2009). *Melampyrum roseum* Maxim. (Scrophulariaceae), a newly recorded genus and species in Taiwan. Taiwania.

[CR8] Chen C-H, Wang C-M (2014). *Antirrhinum orontium* L. (Scrophulariacaee), a newly naturalized genus and species in Taiwan. Coll Res.

[CR9] Christenhusz MJM, Reveal JL, Farjon A, Garden MF, Mill RR, Chase MW (2011). A new classification and linear sequence of extant gymnosperms. Phytotaxa.

[CR10] Chung S-W, Hsu T-C, Jung M-J, Hsiao S-C, Fang W-U (2010). *Lathraea purpurea* (Scrophulariaceae): a new generic record in Taiwan. Taiwan J For Sci.

[CR11] Chung S-W, Hsu T-C, Peng C-I (2010). *Phacellanthus* (Orobanchaceae), a newly recorded genus in Taiwan. Bot Stud.

[CR12] Cronquist A (1968). The evolution and classification of flowering plants.

[CR45] De Smet Y, Granados Mendoza C, Wanke S, Goetghebeur P, Samain M-S (2015). Molecular phylogenetics and new (infra) generic classification to alleviate polyphyly in tribe Hydrangeeae (Cornales: Hydrangeaceae). Taxon.

[CR13] Herbert J (2005). New combinations and a new species in *Morella* (Myricaceae). Novon.

[CR14] Hsieh C-F (2003) Composition, endemism and phytogeographical affinities of the Taiwan flora. In: Editorial Committee of the Flora of Taiwan (ed) Flora of Taiwan, vol 6, 2nd edn. Department of Botany, National Taiwan University, Taipei, pp 1–14

[CR15] Hsu TC, Chen ZH (2017). *Scleromitrion sirayanum* (Rubiaceae: Spermacoceae), a new species of the *Hedyotis-Oldenlandia* complex in Taiwan. Taiwania.

[CR16] Hsu T-W, Chiang T-Y, Chiang Y-C (2016). *Peplidium maritimum* (L.f.) Asch. (Phrymaceae), a new record in the Flora of Taiwan. Taiwan J Biodivers.

[CR17] Hsu TW, Chiang TY, Huang CC (2016). Two newly naturalized species in Taiwan, *Rnellia squarrosa* (Fenzl) Schaffnit and *Micranthemum micrathemoides* (Nutt.) Wettst. Taiwan J Biodivers.

[CR18] Hsu T-W, Kono Y, Chiang T-Y, Peng C-I (2011). *Ypsilandra* (Melanthiaceae: Liliaceae sensu lato), a new generic record for Taiwan. Bot Stud.

[CR19] Huang TC, Huang SF, Hsieh TH (1994). The flora of Tungshatao (Pratas Island). Taiwania.

[CR20] Huang T-C (1994) Preface of Volumen One. In: Editorial Committee of the Flora of Taiwan (ed) Flora of Taiwan, vol 1, 2nd edn. Department of Botany, National Taiwan University, Taipei, pp v–vi

[CR21] Huguet V, Gouy M, Normand P, Zimpfer JF, Fernandez MP (2005). Molecular phylogeny of Myricaceae—a reexamination of host-symbiont specificity. Mol Phylogenet Evol.

[CR22] Jung M-J, Ku S-M, Peng C-I (2011). *Oldenlandiopsis* Terell & W H. Lewis (Rubiaceae), a newly recorded genus in Taiwan. Taiwania.

[CR23] Jung M-J, Liao G-I, Kuoh C-S (2005). *Phryma leptostachya* (Phrymaceae), a new family record in Taiwan. Bot Bull Acad Sinica.

[CR24] Kanis A, Crisp MD, Orchard AE (1999) Classification, phylogeny and the flora of Australia. In: Orchard AE (ed) Flora of Australia, vol 1 Introduction. CSIRO Publishing, Canberra, pp 125–147

[CR25] Ko CJ (2004) A taxonomic and distributional study of seagrasses in Taiwan. Master thesis, National Sun Yat-sen University

[CR500] Les DH, Crawford DJ (1999). *Landoltia* (Lemnaceae), a new genus of duckweeds. Novon.

[CR26] Li C-Y, Wang C-M (2012). *Anoda cristata* (L.) Schltdl. (Malvaceae), a newly naturalized plant in Taiwan. Quart J For Res.

[CR27] Liang Y-S, Jung M-J, Wu S-C, Kao Y-C, Wang J-C (2011). *Stemodia* L. (Scrophulariaceae), a newly naturalized genus in Taiwan. Taiwania.

[CR28] Lin HJ, Hsieh LY, Liu PJ (2005). Seagrasses of Tongsha Island, with descriptions of four new records to Taiwan. Bot Bull Acad Sin.

[CR29] Lindqvist C, De Laet J, Haynes RR, Aagesen L, Keener BR, Albert VA (2006). Molecular phylogenetics of an aquatic plant lineage, Potamogetonaceae. Cladistics.

[CR30] Ling C-J, Chen C-F (2013). *Mitracarpus hirtus* (L.) DC. (Rubiaceae), a newly naturalized plant in Taiwan. Taiwan J Biodivers.

[CR31] Liu B, Ye J, Liu S, Wang Y, Yang Y, Lai Y, Zeng G, Lin Q (2015). Families and genera of Chinese angiosperms: a synopsis classification based on APG III. Biodivers Sci.

[CR32] Lu Y, Ran J-H, Guo D-M, Yang Z-Y, Wang X-Q (2014). Phylogeny and divergence times of gymnosperms inferred from single-copy nuclear genes. PLoS ONE.

[CR33] Luebert F, Cecchi L, Frohlich MW, Gottschling M, Guilliams CM, Hasenstab-Lehman KE, Hilger HH, Miller JS, Mittelbach M, Nazaire M, Nepi M, Nocentini D, Ober D, Olmstead RG, Selvi F, Simpson MG, Sutory K, Valdes B, Walden GK, Weigend M (2016). Familial classification of the Boraginales. Taxon.

[CR34] Maletic JI, Marcus A (2000) Data cleansing: beyond integrity analysis proceedings of the 2000 conference on information quality, 200–209. MIT, Cambridge

[CR35] Manns U, Anderberg AA (2009). New combinations and names in *Lysimachia* (Myrsinaceae) for species of *Anagallis*, *Pelletiera* and *Trientalis*. Willdenowia.

[CR36] Mayr E (1981). Biological classification: toward a synthesis of opposing methologies. Science.

[CR37] Nakamura K, Chung S-W, Kokubugata G, Denda T, Yokota M (2006). Phylogenetic systematics of the monotypic genus *Hayataella* (Rubiaceae) endemic to Taiwan. J Plant Res.

[CR38] Neupane S, Dessein S, Wikström N, Lewis PO, Long C, Bremer B, Motley TJ (2015). The *Hedyotis-Oldenlandia* complex (Rubiaceae: Spermacoceae) in Asia and the Pacific: phylogeny revisited with new generic delimitations. Taxon.

[CR39] Patterson DJ, Cooper J, Kirk PM, Pyle RL, Remsen DP (2010). Names are key to the big new biology. Trends Ecol Evol.

[CR40] Patterson DJ, Egloff W, Agosti D, Eades D, Franz N, Hagedorn G, Rees JA, Remsen DP (2014). Scientific names of organisms: attribution, rights, and licensing. BMC Res Notes.

[CR41] Reveal JL (1993) Flowering plant families: an overview. In: Flora of North America Editorial Committee (ed) Flora of North America North of Mexico, vol 1. Oxford University Press, New York, pp 294–330

[CR42] Ruggiero MA, Gordon DP, Orrell TM, Bailly N, Bourgoin T, Brusca RC, Cavalier-Smith T, Guiry MD, Kirk PM (2015). A higher level classification of all living organisms. PLoS ONE.

[CR43] Shao K-T, Lai K-C, Lin Y-C, Chen L-S, Li H-Y, Hsu C-H, Lee H, Hsu H-W, Mai G-S (2013). Experience and strategy of biodiversity data integration in Taiwan. Data Sci J.

[CR44] Shao K-T, Peng C-I, Wu W-J (eds) (2008) Taiwan species diversity II. Species checklist. Forestry Bureau, Council of Agriculture, Executive Yuan, Taipei

[CR46] The Angiosperm Phylogeny Group (1998). An ordinal classification for the families of flowering plants. Ann Mo Bot Gard.

[CR47] The Angiosperm Phylogeny Group (2003). An update of the Angiosperm Phylogeny Group classification for the orders and families of flowering plants: APG II. Bot J Linn Soc.

[CR48] The Angiosperm Phylogeny Group (2009). An update of the Angiosperm Phylogeny Group classification for the orders and families of flowering plants: APG III. Bot J Linn Soc.

[CR49] The Angiosperm Phylogeny Group (2016). An update of the Angiosperm Phylogeny Group classification for the orders and families of flowering plants: APG IV. Bot J Linn Soc.

[CR50] Thulin M, Moore AJ, El-Seedi H, Larsson A, Christin PA, Edwards EJ (2016). Phylogeny and generic delimitation in Molluginaceae, new pigment data in Caryophyllales, and the new family Corbichoniaceae. Taxon.

[CR51] Trias-Blasi A, Baker WJ, Haigh AL, Simpson DA, Weber O, Wilkin P (2015). A genus-level phylogenetic linear sequence of monocots. Taxon.

[CR52] Wang C-M, Chang K-C, Chen C-H (2016). Two newly naturalized plant species in Taiwan: *Fumaria parviflora* Lam. and *Nelsonia canescens* (Lam.) Spreng. Taiwan J For Sci.

[CR53] Wang C-M, Chen C-H (2013). *Digera muricata* (L.) Mart. (Amaranthaceae), a newly naturalized genus and species in Taiwan. Taiwania.

[CR54] Wang X-Q, Ran J-H (2014). Evolution and biogeography of gymnosperms. Mol Phylogenet Evol.

[CR55] Wearn JA, Chase MW, Mabberley DJ, Couch C (2013). Utilizing a phylogenetic plant classification for systematic arrangements in botanic gardens and herbaria. Bot J Linn Soc.

[CR56] Wu Y-H, Chao C-T, Tseng Y-H (2015). *Eurya lui* (Pentaphylacaceae). Quart J For Res.

[CR57] Wu S-H, Yang T-YA, Teng Y-C, Chang C-Y, Yang K-C, Hsieh C-F (2010). Insights of the latest naturalized flora of Taiwan: change in the past 8 years. Taiwania.

[CR58] Yamashita J, Tamura MN (2004). Phylogenetic analyses and chromosome evolution in Convallarieae (Ruscaceae sensu lato), with some taxonomic treatments. J Plant Res.

[CR59] Yang Y-P, Yen S-H, Lin C-K (2001). Illustrated guide to aquatic plants of Taiwan.

